# miR-424-5p regulates cell proliferation and migration of esophageal squamous cell carcinoma by targeting SIRT4

**DOI:** 10.7150/jca.50587

**Published:** 2020-09-01

**Authors:** Ying Cui, Jiani Yang, Yibing Bai, Yanqiao Zhang, Yuanfei Yao, Tongsen Zheng, Chao Liu, Feng Wu

**Affiliations:** 1 Department of Radiation Oncology, Harbin Medical University Cancer Hospital, Harbin, China.; 2 Translational Medicine Research and Cooperation Center of Northern China, Heilongjiang Academy of Medical Sciences, Harbin, China.; 3 Department of Gastrointestinal Medical oncology, Harbin Medical University Cancer Hospital, Harbin, China.; 4 Department of Gastroenterology, The First Affiliated Hospital of Harbin Medical University, Harbin, China.

**Keywords:** esophageal squamous cell carcinoma, ESCC, miR-424-5p, SIRT4, *in vivo*

## Abstract

**Objective:** The present research is aimed to elucidate the expression patterns of miR-424-5p and its role in tumorigenesis and progression of esophageal squamous cell carcinoma (ESCC).

**Methods:** Both starBase and TCGA were utilized to assess miR-424-5p expression status in ESCC. The endogenous mRNA expression levels of miR-424-5p in ESCC and normal esophagus cell lines were detected by qRT-PCR. CCK8 and colony-forming assays were applied to determine the effects of miR-424-5p on ESCC proliferation. Transwell migration and wound healing assays were carried out to observe the changes of ESCC cell mobility after miR-424-5p mimic or inhibitor transfection. Impact of miR-424-5p on malignancy growth *in vivo* was further verified in a mouse xenograft model. The regulatory relationships between miR-424-5p and SIRT4 were validated by dual luciferase reporter assay, qRT-PCR and Western blot.

**Results:** miR-424-5p expression was found upregulated in ESCC. miR-424-5p overexpression dramatically facilitated ESCC cells proliferation and migration capacity *in vitro*, while downregulation of miR-424-5p displayed the opposite trend. Inhibition of xenograft tumor growth was further evidenced *in vivo*. Moreover, SIRT4 was confirmed to be a specific target gene of miR-424-5p in ESCC and negatively modulated by miR-424-5p. Finally, SIRT4 overexpression strongly rescued the promoting influence of miR-424-5p on the proliferative and migratory capacity of ESCC cells.

**Conclusions:** miR-424-5p had tumor promoting functions in proliferation and migration of ESCC by targeting SIRT4, suggesting that miR-424-5p may serve as a potential diagnostic biomarker and manipulation of miR-424-5p/SIRT4 axis could provide a novel therapeutic strategy for further ESCC treatment.

## Introduction

Esophageal carcinoma (EC) is a fatal malignant disease, which ranks the seventh in morbidity and the sixth in mortality worldwide 1. Squamous cell carcinoma and adenocarcinoma are the two dominant pathological types of EC, and adenocarcinoma is more common in Western countries. In China, more than 95% of EC are squamous cell carcinoma 2, which is listed as the third most common malignant tumor and the fourth in mortality 3. In recent years, radical surgery is the principal treatment for esophageal squamous cell carcinoma (ESCC). Simultaneous chemoradiotherapies have become more and more valued as an option for ESCC comprehensive treatment mode 4, while targeted drug treatment and immunotherapy are still developing. However, patients with ESCC are often diagnosed at advanced stage for which curative resection is not achievable, and other therapeutic approaches are not ideal. Hence, new treatment modalities are urgently needed 5. Therefore, there is an imperative necessity to deeply understand underlying molecular mechanisms of ESCC and identify novel potential biomarkers for early diagnosis and effective treatments.

Non-coding RNA (ncRNA), particularly microRNA (miRNA), long-stranded ncRNA (lncRNA) and circular RNA (circRNA) have been reported as functional regulatory molecules involved in the development of many human cancers 6. MicroRNAs (miRNAs) are single-stranded non-coding small RNA segments that operate via sequence-specific interactions with the 3′ untranslated regions (3′UTRs) of mRNA targets to suppress translation and/or promote mRNA decay to regulate gene expression post-transcriptionally 7. Recent studies have shown that miR-424 is involved in the occurrence and development of various kinds of malignant tumors. In prostate cancer, miR-424 targets E3 ubiquitin ligase COP1 to impair its function, thus activating STAT3 which is a key substrate of COP1 and promoting prostate tumor progression 8. In thyroid cancer, miR-424-5p facilitates anoikis resistance and lung metastasis by inactivating Hippo signal transduction 9. Additionally, miR-424-5p exhibits a pivotal impact in cervical 10, ovarian 11, hepatocellular 12, leukemia 13, breast cancer 14 and so on.

SIRT4, a member of SIRT family, has been well recognized as a highly conserved nicotinamide adenine dinucleotide (NAD^+^)-dependent enzyme. Recently, growing evidence has suggested that SIRT4 plays essential roles in longevity, oxidative stress, and metabolism 15. SIRT4 also functions as a tumor suppressor in many types of malignant tumors, such as lung cancer 16, colorectal cancer, etc 17, 18. Yujiro Nakahara et al. revealed that SIRT4 inhibition was correlated with distant recurrence in ESCC patients, and ESCC cell migration 19.

Here, we observed that miR-424-5p could accelerate proliferation and migration of ESCC in our *in vitro* and *in vivo* experiments. In addition, we identified SIRT4 as a direct target of miR-424-5p. Rescue assays further illustrated that miR-424-5p indeed manipulated proliferation and migration of ESCC cells through SIRT4. Thereby, miR-424-5p can be deemed as a potential biomarker for ESCC, and the miR-424-5p/SIRT4 axis may represent a promising choice for further ESCC therapeutic strategy.

## Methods and materials

### Cell lines and Cell culture

The normal human esophagus epithelial cell line HEEC, and ESCC cell lines, including EC-9706, Eca-109, KYSE-150, TE-1, were purchased from Shanghai Zishi Biotechnology Co. Ltd. (Shanghai, China). The cells were preserved in RPMI-1640 (Gibco, USA) or Dulbecco's modified Eagle's medium (DMEM; Gibco, USA) with 10 % fetal bovine serum (FBS, Sciencell, USA) plus 1% penicillin G/streptomycin at 37 °C in a humidified air containing 5% CO2.

### Plasmids, RNA transfection and lentiviral transduction

The cells growing at logarithmic phase were planted into 6-well plates to achieve a density of 60%. Transient transfection with plasmids pcDNA3.1 vector or SIRT4-Flag (Addgene, #151245), miR-424-5p mimic, miR-424-5p inhibitor, or matched control (mimic/inhibitor NC; GenePharma, shanghai, China) were performed as indicated in the figures by using Lipofectamine 2000 (Thermo Fisher Scientific, USA) per the manufacturer's instruction. 6 h after transfection, each well was replaced with medium containing 10% FBS for further culture. After 48h, the expression of miR-424-5p or SIRT4 was detected by qRT-PCR and/or Western blot.

In order to establish stable miR-424-5p knockdown cells, Eca-109 was sowed into 6-well plates and infected with hsa-miR-424-5p shRNA lentiviral plasmid and negative control vector obtained from Genechem (Shanghai, China). Puromycin was used to select infected cells. After 10 days, stable miR-424-5p knockdown cell lines were confirmed. The sequences of shRNA and negative control were as table 1.

### qRT-PCR analysis

Total RNA samples were extracted from cultured cell lines with TRIzol (Thermo fisher Scientific, Waltham, MA, USA). The Reverse Transcriptase kit (Roche, Penzberg, Germany) was employed to reverse transcription for acquiring cDNA, and SYBR Green Master (Roche) was used for RT-PCR to detect the mRNA expression of miR-424-5p and SIRT4. U6 and GAPDH were used as internal controls. Ct values were calculated to analyze the relative expression levels by the 2^-ΔΔCT^ method. Primers utilized in this study were listed in table 2.

### Western blot analysis

Cells in different groups were collected and centrifuged, and the supernatant was transferred to sterile EP tubes. Total protein was lysed with RIPA lysis buffer (Solarbio, Beijing, China) for 30min on ice and the level of protein concentration was measured with BCA assay (Beyotime, Shanghai, China) at 562 nm optical density. Proteins lysates were denatured by SDS-PAGE and inversed to polyvinylidene fluoride (PVDF) membranes (Millipore, USA) which were blocked by 5% skim‐milk for 1h at room temperature. Then membranes were incubated with primary antibodies: SIRT4, β-actin (Proteintech, Wuhan, China) at the temperature of 4°C overnight. The second day, after being washed with PBST for 3 times, membranes were cultivated with secondary antibodies (diluted at 1:4000) at ambient temperature for an hour. Chemiluminescence device was used for color development.

### Cell proliferation

48h after transfection, about 3×10^3^ cells in 100 μl medium were seeded in 96-well plates and cultivated in 10% FBS. Cell counting kit-8 (CCK-8, Dojindo, Kumamoto, Japan) agent was added in each well at the following time points, 0 h, 24 h, 48 h and 72 h (10 μl CCK-8 solution per 100 μl medium). Cell proliferation was determined by measuring the wells at 450 nm, 630 nm optical density.

### Colony-forming assay

Cancer cells in the logarithmic phase were planted in a 6-well plate (1000 per well) at 48 h after transfection and cultured for 11 to 14 days. After PBS wash, colonies were visualized with methanol and 1% crystal violet staining, and then the visible colonies were counted.

### Transwell migration assay

At 48h after transfection, 5×10^5^ cells were added to the upper chamber (Corning Costar Corp, Cambridge, MA) in 200 μl of serum-free RPMI-1640 or DMEM medium. 700 μl of medium blending 20% FBS was added in the lower chambers. After 24 h incubation, migrating cells fixed in the bottom were stained with methanol and 1% crystal violet for 20 min and enumerated.

### Wound healing assay

Cells were sowed into 6-well plates in medium containing 10% FBS and cultured to 70%-80% confluence and allowed to attach for 24 h. The cells were scraped with a pipette tip (20 μl) to generate a scrap wound, and replaced with medium containing 5% FBS. The distance of wounds was measured at 0 h and 24 h after scraping for wound healing analysis.

### miRNA target prediction analysis

Four online prediction databases including TargetScan (http://www.targetscan.org), miRDB (http://www.mirdb.org/), mirDIP (http://ophid.utoronto.ca/mirDIP/) and miRWalk (http://mirwalk.umm.uni-heidelberg.de/) were utilized to analyze potential miRNA targets and predict sites that bind to miR-424-5p. Venn diagrams of gene expression were plotted by applying Bioinformatics & Evolutionary Genomics online analysis software (http://bioinformatics.psb.ugent.be/webtools/Venn/).

### Luciferase reporter gene assay

~200bp DNA fragment covering the predicted miR-424-5p binding site (WT) on the target gene was inserted into pmirGLO vector. Plasmid with mutated binding site (MT) was also constructed as a control. Co-transfection was performed based on four experimental groups (NC + SIRT4-WT, mimic + SIRT4-WT, NC + SIRT4-MT, and mimic + SIRT4-MT), and the Luciferase Reporter Assay Kit was used to detect luciferase activity as per the manufacturer's protocol.

### Mouse xenograft tumor model

Female BALB/c-nude mice (5 to 6 weeks old) were purchased from the Charles River Laboratories (Beijing, China). Ten mice were randomly divided into two groups of five (anti-miR-424-5p and anti-NC). Stable Eca-109 cells (5 × 10^6^) were injected into each mouse's left underarm. One week after inoculation, measurement of the volume (0.5 × length × width^2^) of the tumors was started with vernier caliper every 3 days for 27 days for tumor growth curve plotting. At the conclusion of the experiment, the mice were sacrificed, and the tumors were photographed and weighed. All animal researches were approved by the Ethics Committee for Animal Experimentation of Harbin Medical University.

### Statistical analysis

All the experiments were performed in triplicate and presented as the mean ± SD when applicable. Statistical data were analyzed with GraphPad Prism version 7.03 (GraphPad Software Inc, San Diego, CA, USA). Two-way ANOVA or student's *t*-test was selected to compare differences between groups, and Wilcoxon rank sum test was applied in two independent samples. *P* < 0.05 was considered typically statistically significant.

## Results

### miR-424-5p is upregulated in ESCC

**Figure 1:**
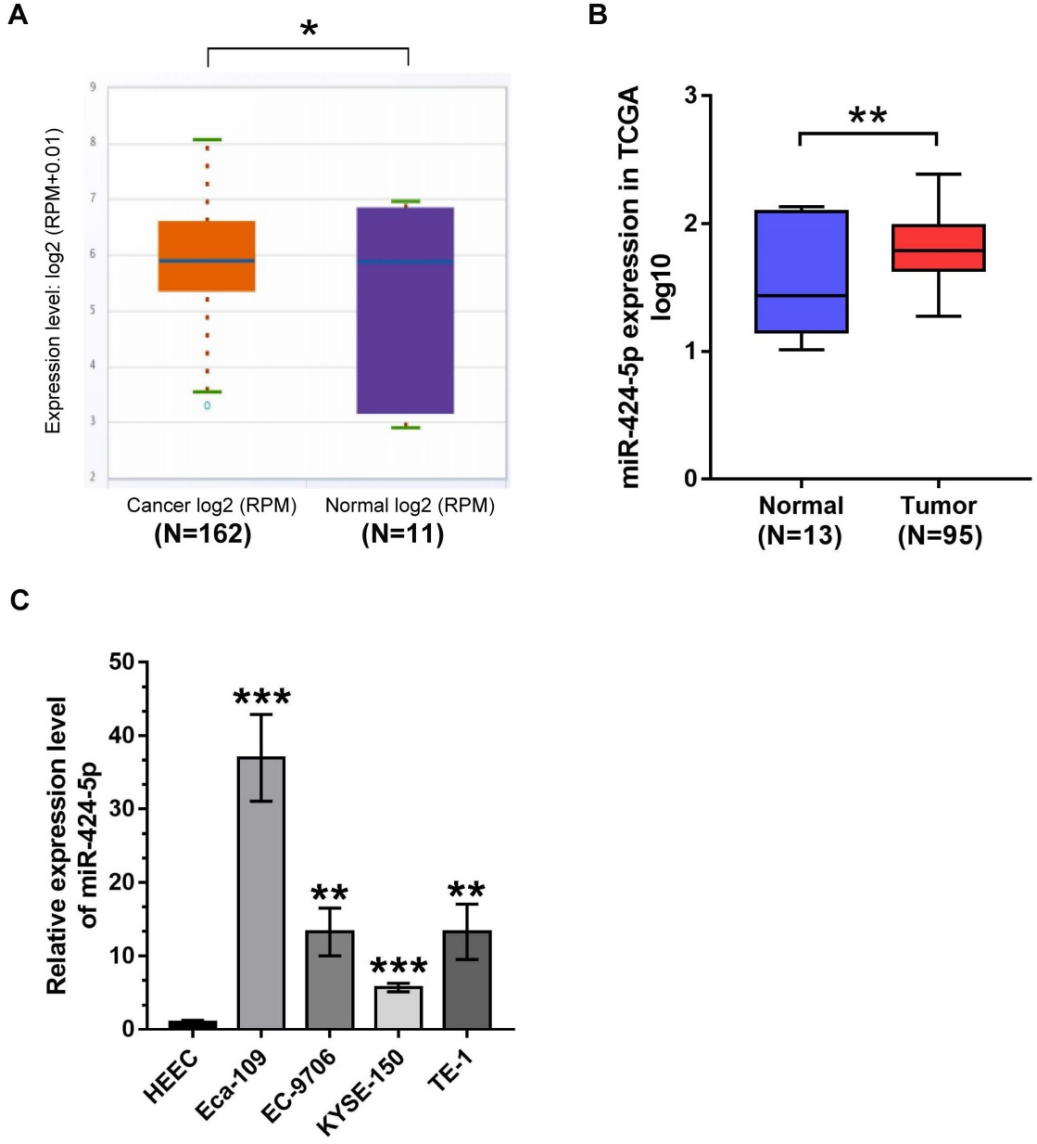
** The expression of miR‐424‐5p is increased in ESCC.** (**A**) Comparison of mir-424-5p expression between ESCC tissues and normal esophagus specimens in starBase. P = 0.012. (**B**) Upregulated expression of miR-424-5p between ESCC patients (n = 95) and normal esophagus tissues (n = 13) by bioinformatics analysis from TCGA was shown as a log10-fold change, student's *t*-test, *P* = 0.00955. (**C**) Endogenous miR-424-5p expression status was quantified in ESCC cells (Eca-109, EC-9706, KYSE-150, TE-1) compared with normal human esophagus epithelial cell line (HEEC) by qRT-PCR. Data are presented as mean ± SD of three separate experiments. *P < 0.05, **P < 0.01, ***P < 0.001.

We first attempted to clarify the expression status of miR-424-5p in ESCC patients archived in online databases including starBase (http://starbase.sysu.edu.cn/index.php) and The Cancer Genome Atlas (TCGA) database (https://portal.gdc.cancer.gov/legacy-archive/search/f). As presented in Fig. 1A, miR-424-5p was significantly elevated in 162 ESCC patients compared with 11 normal esophagus samples from starBase (*P* = 0.012). Meanwhile, bioinformatics analysis from TCGA further confirmed similar conclusion that miR-424-5p expression was increased in ESCC specimens (Fig. 1B). Consistently, miR-424-5 levels in four ESCC cell lines (Eca-109, EC-9706, KYSE-150, TE-1) were all significantly higher than in normal human esophagus epithelial cell line (HEEC), ranging from ~5-fold to ~35-fold higher (Fig. 1C). These data clearly demonstrate that miR-424-5p is upregulated in ESCC and is a potential oncogene.

### miR-424-5p facilitates ESCC proliferation and migration *in vitro*

**Figure 2:**
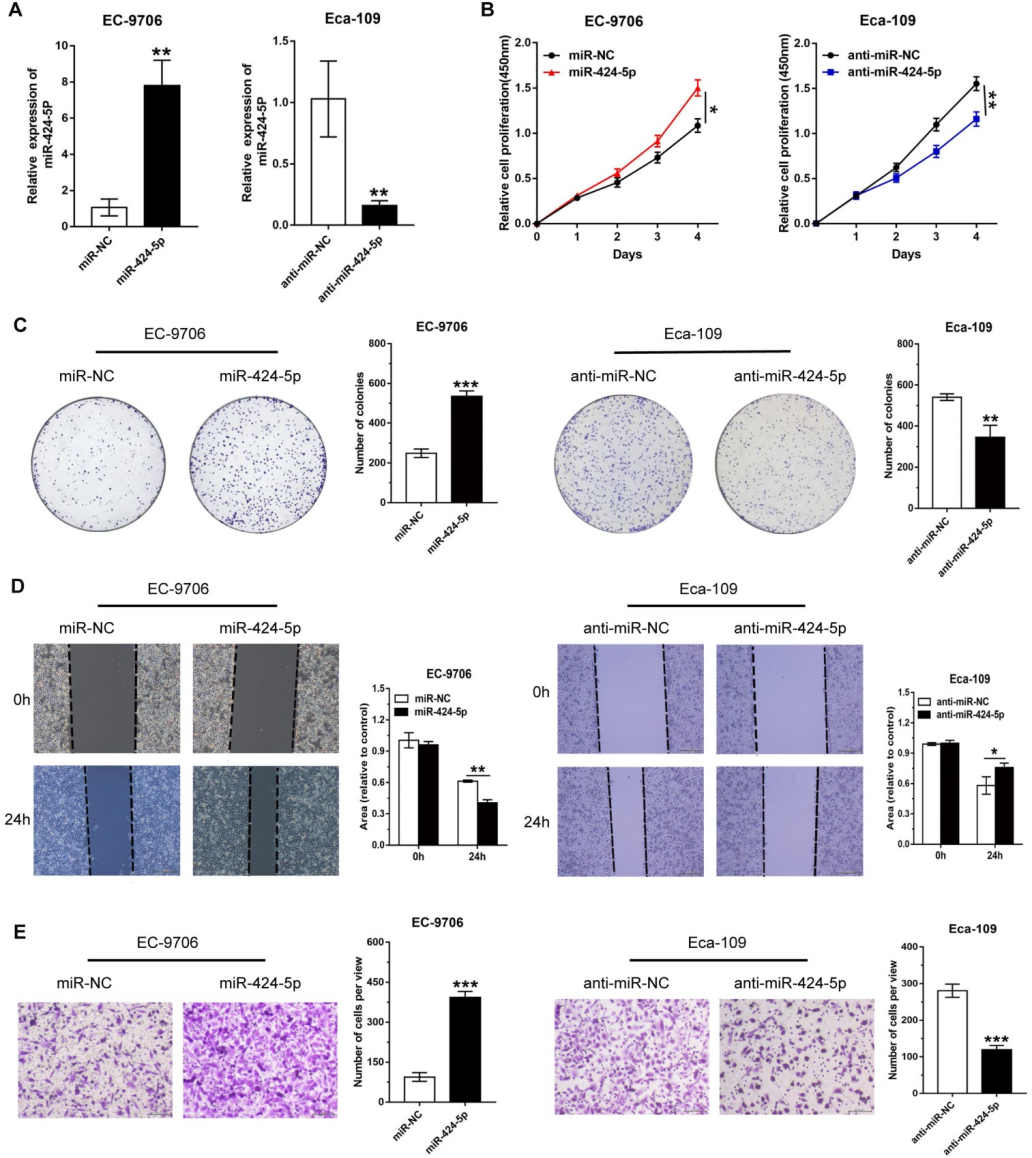
** miR‐424‐5p regulates ESCC cells proliferation and migration *in vitro*.** (**A**) The miR‐424‐5p overexpression or knockdown efficiency was validated by qRT-PCR after EC-9706/Eca-109 were transfected with miR‐424‐5p‐mimic or inhibitor for 48 hours. (**B**) Cell viabilities were determined by CCK-8 assay at 0, 1, 2, 3, 4 days in miR-424-5p overexpressed EC-9706 and miR-424-5p silenced Eca-109. (**C**) Colony-forming assay was performed to assess colony formation in ESCC cells with miR-424-5p overexpression or knockdown. (**D**) Wound healing and (**E**) Transwell migration assays were employed to evaluate cells migration ability in miR-424-5p‐overexpression and miR-424-5p-knockdown EC-9706/Eca-109. Data are presented as mean ± SD of three separate experiments. *P < 0.05, **P < 0.01, ***P < 0.001.

According to the miR‐424‐5p expression status in ESCC cells, ECA-109 was selected to silence miR‐424‐5p, while EC-9706 was chosen to upregulate miR‐424‐5p in subsequent experiments. Compared with matched controls, miR-424-5p expression was conspicuously increased after miR-424-5p mimic transfection in EC-9706 while reduced after transfection with inhibitor in Eca-109 (Fig. 2A).

To illustrate the potential biological functions of miR-424-5p in ESCC, we then performed various tumorigenic phenotyping experiments. The CCK8 assay revealed that increase in miR-424-5p expression typically expedited cell proliferation in EC-9706, while anti-miR-424-5p transfection attenuated growth of Eca-109 cells (Fig. 2B). Similarly, the effects of miR-424-5p or anti-miR-424-5p were consistently observed in the colony-forming assay (Fig. 2C). In addition, we conducted wound healing and Transwell analyses to evaluate the roles of miR-424-5p in ESCC cells migration. Wound healing assay revealed that overexpression of miR-424-5p upregulated the migration quantity of EC-9706, and miR-424-5p knockdown significantly delayed the wound closure in Eca-109 (Fig. 2D). As shown in Fig. 2E, Transwell migration assay further demonstrated that miR-424-5p overexpression accelerated EC-9706 passing through the filter membranes. On the contrary, silence of miR-424-5p restrained the migration capability of Eca-109. In short, these results indicate that miR-424-5p has a promoting role in ESCC cell proliferation and migration.

### miR-424-5p knockdown inhibits ESCC growth *in vivo*

**Figure 3:**
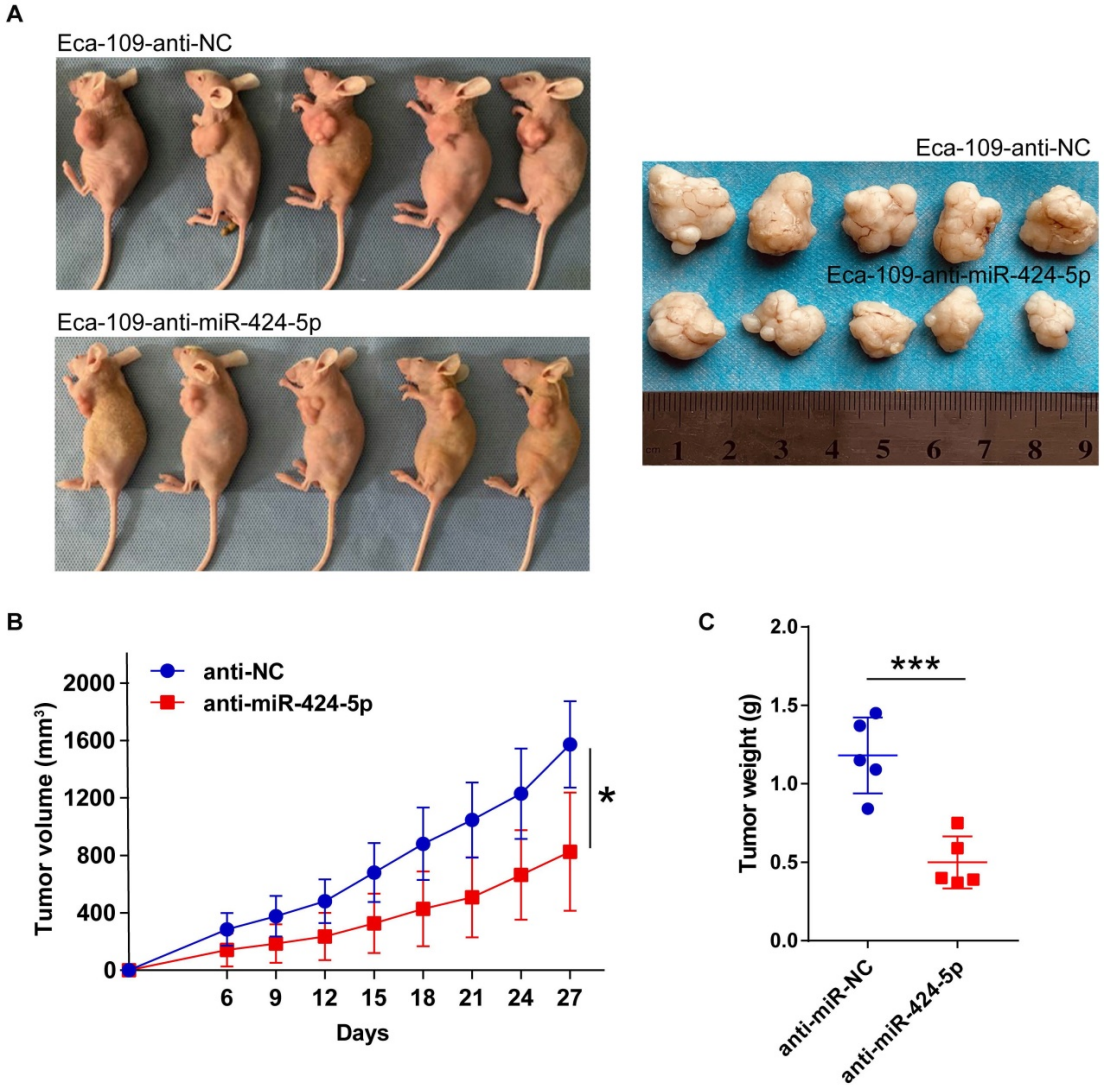
** miR-424-5p knockdown restrains ESCC growth *in vivo*.** (**A**) Representative image of tumors collected from anti-miR-424-5p and anti-NC groups of BALB/c nude mice on day 27 after subcutaneous injection with 5×10^6^ Eca-109 cells. (**B**) Xenograft tumor volumes in each group (n = 5) were recorded every 3 days and tumor growth curve was plotted. (**C**) Xenograft tumor weights between anti-miR-424-5p and anti-NC groups were compared by plotting a scatter diagram. Data are presented as mean ± SD. *P < 0.05, **P < 0.01, ***P < 0.001.

We next explored whether miR-424-5p affects neoplasm formation in nude mice. Ten mice were randomly assigned into two groups receiving 5×10^6^ Eca-109 cells stably expressing anti-miR-424-5p and anti-NC, respectively, in left underarm subcutaneously. Tumor volume was measured starting on day 6 after injection and the measurement was carried out every 3 days for 27 days. As compared control group, inhibition of miR-424-5p clearly slowed down the tumor growth (Fig. 3B). At the conclusion of the experiment, although all the mice developed xenograft tumors, the final sizes and weights of the tumors formed from anti-miR-424-5p expressing cells were more than 2-fold smaller than those formed from control cells (Fig. 3A & C). These findings further support the tumor suppressor role of miR-424-5p in ESCC as observed in our *in vitro* experiments.

### SIRT4 is a direct target of miR-424-5p in ESCC cells

**Figure 4:**
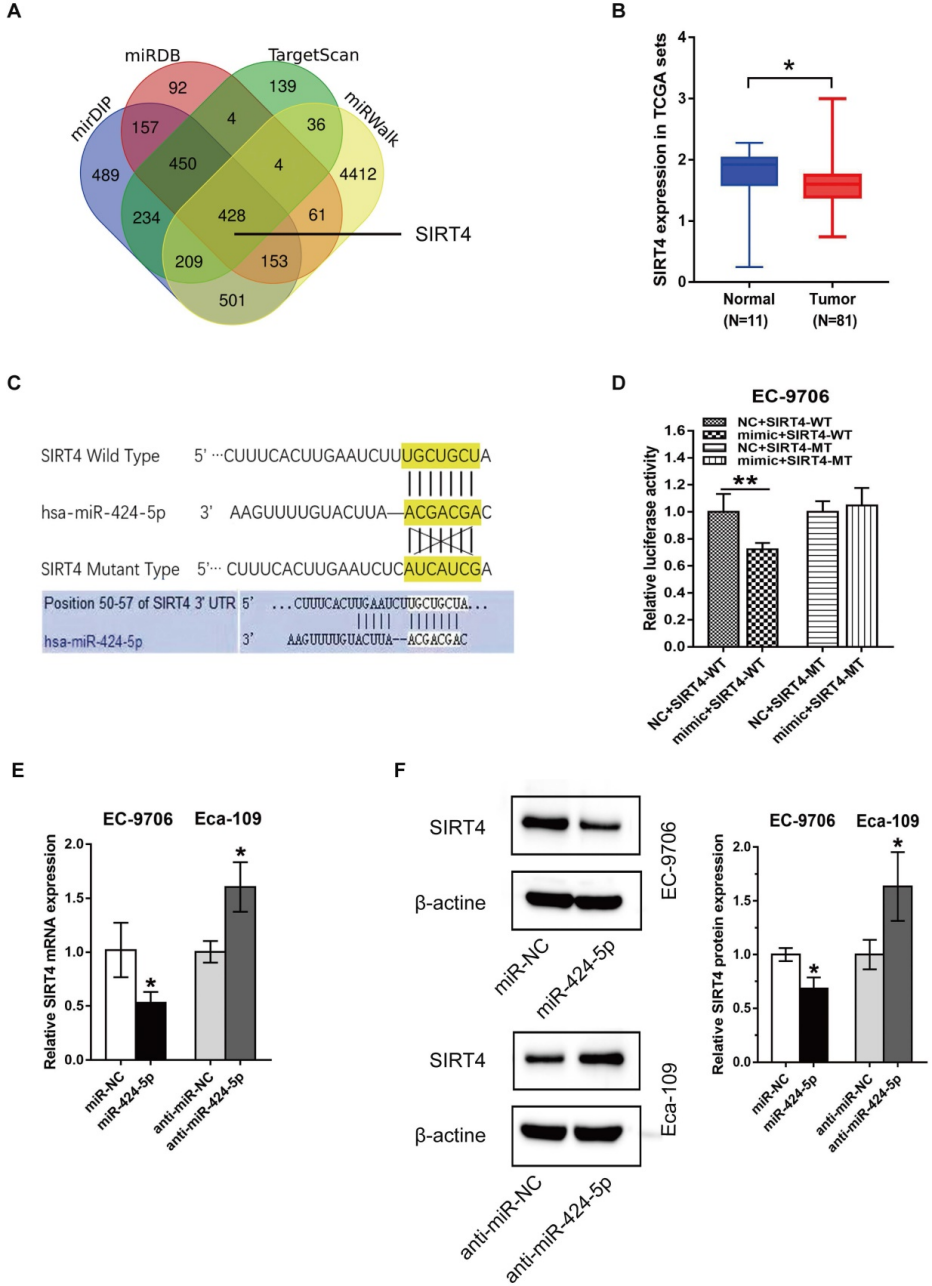
** miR-424-5p directly targets SIRT4 in ESCC.** (**A**) Venn analysis of miR-424-5p target gene prediction through four bioinformatics databases: TargetScan, miRDB, mirDIP and miRWalk. (**B**) SIRT4 expression between normal esophagus tissues and ESCC samples was contrasted using TCGA database. The wilcoxon rank sum test, *p* = 0.021. (**C**) Predicted binding sites of miR-424-5p and the 3′-UTR of SIRT4 mRNA (wild type and mutant for pmirGlo constructs) were highlighted in yellow. (**D**) Luciferase activity in EC-9706 transfected with SIRT4 3'-UTR-WT or SIRT4 3'-UTR-MT were observed between miR-424-5p mimic group and NC group. (**E**) qRT-PCR and (**F**) Western blot analyses were applied to measure mRNA and protein levels of SIRT4 in EC-9706 and Eca-109 transfected with miR-424-5p inhibitor or mimic. Data are presented as mean ± SD of three separate experiments. *P < 0.05, **P < 0.01, ***P < 0.001.

To gain further insight of miR-424-5p in ESCC, we employed four online bioinformatics databases including TargetScan, miRDB, mirDIP and miRWalk to identify potential target genes of miR-424-5p. The Venn analysis revealed that a total of 428 genes were present in all these four databases (Fig. 4A). Given the facts that SIRT4 expression is often downregulated in most malignant cancers 16-18 and correlated with a poor clinical outcome in ESCC 19, and SIRT4 is among the 428 genes mentioned above with high matching score (Fig. 4A), we focused on validation of SIRT4 in our subsequent experiments.

**Figure 5:**
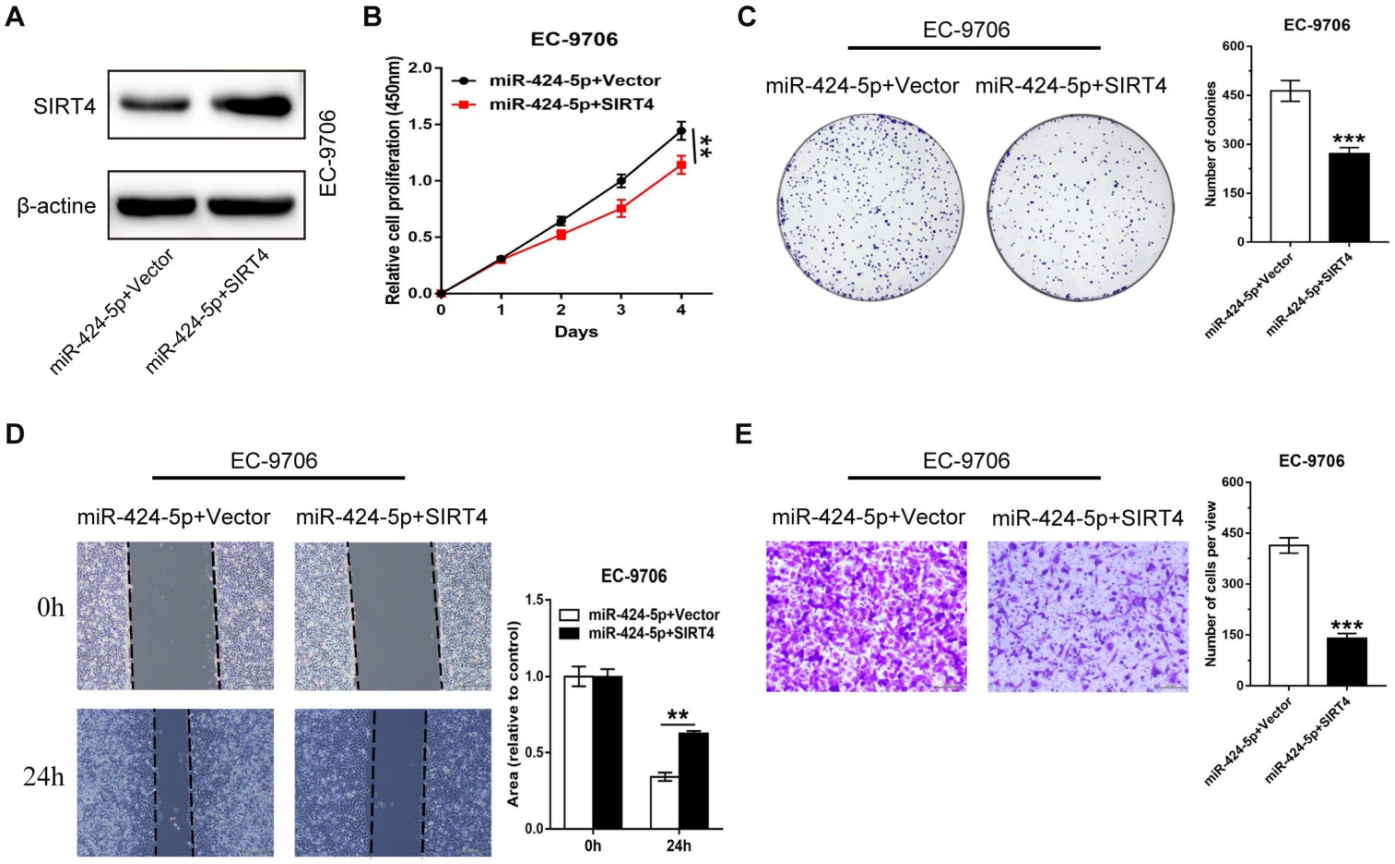
** miR-424-5p induced proliferation and migration promotion are rescued by overexpression of SIRT4.** (**A**) The expression level of SIRT4 protein in EC-9706 overexpressing miR-424-5p was detected by western blot after the cells were transfected with SIRT4 or empty vector. (**B**) CCK-8 and (**C**) Colony-forming assays were performed to examine the effects of SIRT4 on proliferative ability of miR-424-5p overexpressing EC-9706 cells. (**D**) Wound healing assay and (**E**) Transwell assay were conducted to determine the roles of SIRT4 on the cell migration of EC9706-miR-424-5p compared with EC9706-NC. Data are presented as mean ± SD of three separate experiments. *P < 0.05, **P < 0.01, ***P < 0.001.

We checked the expression level of SIRT4 in ESCC samples and normal tissues retrieved from TCGA which contains 81 ESCC tissues and 11 adjacent normal esophagus tissues. As shown in Fig. 4B, SIRT4 was low expressed in ESCC (p = 0.021), which is opposite to miR-424 expression (Fig. 1B). Figure 4C showed predicted binding site of miR-424-5p on the 3'UTR of SIRT4 mRNA, based on which we constructed pmirGlo plasmids containing wild-type (WT) and mutant (MT) sequences for dual-luciferase reporter assay. As shown in Fig. 5D, miR-424-5p mimic refrained luciferase activity in EC-9706 cells transfected with SIRT4 3'-UTR-WT construct but did not affect that of SIRT4 3'-UTR-MT (Fig. 4D), suggesting that the WT binding site is responsible for miR-424-5p inhibition of luciferase activity. To further validate the effects of miR-424-5p on SIRT4, we then tested the expression of SIRT4 at both mRNA and protein levels in response to miR-424-5p mimic or inhibitor treatments. As expected, transfection with miR-424-5p mimic significantly downregulated SIRT4 mRNA and protein expression in EC-9706 cells, and in contrast, anti-miR-424-5p transfection in Eca-109 cells displayed opposite effects (Fig. 4E & F). Taken together, these data indicate that SIRT4 is a direct target of miR-424-5p.

### The promoting effect of miR-424-5p on ESCC proliferation and migration is rescued by SIRT4 overexpression

We have shown that miR-424-5p enhanced the proliferation and migration ability of ESCC *in vitro* and *in vivo*, and SIRT4 3'-UTR was a direct target of miR-424-5p. We then attempted to assess if SIRT4 was a critical molecule downstream of miR-424-5p. EC-9706 cells overexpressing miR-424-5p were transfected with SIRT4 plasmids or empty vectors as control. The expression level of SIRT4 protein was increased after EC-9706 cells were co-transfected with SIRT4 and miR-424-5p compared with cells transfected with miR-424-5p alone (Fig. 5A). As CCK‐8 (Fig. 5B) and colony-forming (Fig. 5C) assays indicated, the promoting effect on cell proliferation by miR-424-5p in EC-9706 were significantly receded by ectopic expression of SIRT4. As anticipated, wound healing and Transwell migration analyses also showed that the augmented migratory capabilities by miR-424-5p in ESCC cells were weakened after SIRT4 overexpression (Fig. 5D, E). Collectively, our data provides convincing evidence that SIRT4 is a functional target gene of miR-424-5p.

## Discussion

ESCC, as one of the most invasive malignancies, has a high incidence in Asia 20. A better understanding of the carcinogenic mechanisms of ESCC is critical for developing more effective diagnostic and therapeutic strategies. The regulation of non-coding RNA such as microRNAs has emerged to be an important area of cancer research. MicroRNAs can modulate gene expression through binding to mRNAs and act as a cancer suppressor or oncogene, and therefore have been considered as potential biomarkers and novel therapeutic targets 21, 22. Recently, miR-424-5p has been recognized as an essential influencing factor in a variety of human cancers. Ritu S et al. demonstrated that miR-424 restrained the proliferation and migration of osteosarcoma by upregulating cyclin A2 (CCNA2) 23. miR-424-5p inhibited diversion and invasion of intrahepatic cholangiocarcinoma (ICC) through interacting with ARK5 as reported from the research by Wu JB et al. 24. Li T et al. showed that miR-424-5p also reduced tumor growth in ovarian carcinoma through targeting KIF23, which was silenced by DNA methylation 25. In any case, plenty of studies considered miR-424-5p as an anti-tumor miRNA participating in tumorigenesis and progression of various tumors. Moreover, it can also act as an oncogene in some tumors. Liu XL et al. suggested that miR-424-5p facilitated anorexia and lung metastasis by passivating Hippo signaling in thyroid cancer 9. Additionally, miR-424-5p enhanced the proliferation and metastasis of colorectal cancer through direct inhibition of SCN4B 26. Moreover, miR-424-5p has been identified as an oncogene affecting cell cycle process by directly targeting CADM1 in laryngeal squamous cell carcinoma 27.

In a recent research, Wen, J. et al. illustrated that upregulated miR-424 significantly strengthened ESCC cells proliferation and had a tight connection with unfavorable prognosis in ESCC patients 28. In our study, we clarified that miR-424-5p expression levels in ESCC were significantly higher than normal esophagus tissues or normal cells, which was corroborated with previous studies. Moreover, functional tests (CCK8, colony-forming, wound healing and Transwell) elucidated that miR-424-5p knockdown restricted ESCC proliferation and migration compared with negative control. Similarly, a xenograft model was established for to validate our *in vitro* findings. These results further confirmed the oncogenic role of miR-424-5p in ESCC.

For the purpose of understanding how miR-424-5p promoted malignant phenotypes on ESCC, we utilized four bioinformatics databases (TargetScan, miRBD, mirDIP, and miRWalk) to screen for potential miR-424-5p target genes. Previous data have proved that miR-15b, miR-497 and miR-424 all belong to the miR-16 family which always have semblable factions 23. Interestingly, miRNA-15b managed mitochondrial ROS production and the phenotype associated with aging about SIRT4 29. Meanwhile, miRNA-497 restrained cardiac hypertrophy through targeting SIRT4 30. This led to our hypothesis that miR-424 may also have a potential connection with SIRT4. It's well known that SIRT4 is a potential prognostic biomarker in a multitude of malignancies and enacts a crucial character in regulating cell biology. Wang YS et al. discovered that SIRT4 could inhibit tumorigenesis and advance in HCC through crippling glutamine metabolism and blocking mTOR signaling pathway which based on enhancing the ADP/AMP levels through phosphorylation of AMPK via LKB1 31. Also, SIRT4 overexpression induced cell cycle arrest in G0/G1 phase in thyroid cancer 32. Most importantly, previous studies indicated that down-regulated SIRT4 was linked with unfavorable prognosis and distant recurrence of ESCC 19. In our study, we also found out that SIRT4 expression was lower in ESCC than normal tissues in TCGA database. In line with our bioinformatics result that SIRT4 was predicted as a potential miR-424-5p target in all four databases, our dual luciferase assay confirmed that SIRT4 was indeed a direct target of miR-424-5p. Markedly, the rescue experiments consolidated that SIRT4 is an important downstream target responsible for the oncogenic functions of miR-424-5p in ESCC.

In recent years, SIRT4, as a mitochondrial protein 33, has attracted increasing attention for its role in regulating tumor energy metabolism. SIRT4 was involved in regulating glutamine metabolism by inhibiting glutamate dehydrogenase (GDH) 34, glucose metabolism by inhibiting pyruvate dehydrogenase complex (PDH) 35 and lipid metabolism by targeting malonyl-CoA decarboxylase (MCD) 36. Since we have confirmed miR-424-5p has a tumor promoting function in proliferation and migration of ESCC by targeting SIRT4, we speculate that there may be a close association between miR-424-5p and metabolic reprogramming of ESCC. Testing this hypothesis will be the focus of our future work.

In summary, our study confirmed miR-424-5p as an oncogene for accelerating proliferation and migration of ESCC *in vitro* and *in vivo*, and for the first time we identified SIRT4 as a direct target of miR-424-5p in ESCC. These findings underscore the importance of miR-424-5p and/or miR-424-5p/SIRT4 axis as an effective therapeutic target for treating ESCC.

## Figures and Tables

**Table 1:** The sequences of shRNA and negative control

Primer	Sequence
Hsa-miR-424-5p shRNA	5′- TTCAAAACATGAATTGCTGCTG -3';
shRNA-negative control	5′-TTCTCCGAACGTGTCACGT-3'.

**Table 2:** List of primers

Primer	Sequence
SIRT4-F	5′-GGTCAGTGCGGGCATAAA-3′;
SIRT4-R	5′-TGCTCGAAAGCCTCCATT-3′;
miR-424-5p-F	5′-GCGGCCAGCAGCAATTCATG-3′;
miR-424-5p-R	5′-CAGCCACAAAAGAGCACAAT-3′;
U6-F	5′-GCTTCGGCAGCACATATACTAAAAT-3′;
U6-R	5′-CGCTTCACGAATTTGCGTGTCAT-3′;
GAPDH-F	5′-CATGTTCGTCATGGGTGTGAA-3′;
GAPDH-R	5′-GGCATGGACTGTGGTCATGAG-3′.
